# Flexible Coupling of Respiration and Vocalizations with Locomotion and Head Movements in the Freely Behaving Rat

**DOI:** 10.1155/2016/4065073

**Published:** 2016-07-25

**Authors:** Joseph Andrews Alves, Barbara Ciralli Boerner, Diego Andrés Laplagne

**Affiliations:** Brain Institute, Federal University of Rio Grande do Norte, Avenida Nascimento de Castro 2155, 59056-450 Natal, RN, Brazil

## Abstract

Quadrupedal mammals typically synchronize their respiration with body movements during rhythmic locomotion. In the rat, fast respiration is coupled to head movements during sniffing behavior, but whether respiration is entrained by stride dynamics is not known. We recorded intranasal pressure, head acceleration, instantaneous speed, and ultrasonic vocalizations from male and female adult rats while freely behaving in a social environment. We used high-speed video recordings of stride to understand how head acceleration signals relate to locomotion and developed techniques to identify episodes of sniffing, walking, trotting, and galloping from the recorded variables. Quantitative analysis of synchrony between respiration and head acceleration rhythms revealed that respiration and locomotion movements were coordinated but with a weaker coupling than expected from previous work in other mammals. We have recently shown that rats behaving in social settings produce high rates of ultrasonic vocalizations during locomotion bouts. Accordingly, rats emitted vocalizations in over half of the respiratory cycles during fast displacements. We present evidence suggesting that emission of these calls disrupts the entrainment of respiration by stride. The coupling between these two variables is thus flexible, such that it can be overridden by other behavioral demands.

## 1. Introduction

Mammalian respiration is a fundamentally rhythmic motor program, linking cycles of inhalation-exhalation to conform an oscillation of flow in the airways. Homeostatic and behavioral demands flexibly modulate the rate of this oscillation. In rats, respiratory rate varies from ~1 Hz during sleep, through ~3 Hz during quiet waking, up to 11 Hz during active behavior [1–4]. Discrete events like emitting a vocalization or uttering a word [3–6] or apneic reflexes to noxious smells [7] can also dramatically affect the duration of a respiratory cycle. Actively behaving rats perform other motor programs with marked rhythmicity. While staying in place rats can engage in a stereotyped multisensory exploration of the environment known as sniffing behavior [1, 8]. During sniffing, respiration, whisking, and head position all oscillate with rates in the 5–11 Hz. Moreover, all of them synchronize into a common cycle, with inhalation, vibrissae protraction, and head approach being followed by exhalation, vibrissae retraction, and head withdrawal [1].

Locomotion is rhythmic too, from the chaining of step cycles or strides. As quadrupeds increase their locomotion speed, they transition through three main gaits: walking, trotting, and galloping [9]. Rats switch from walking to trotting at ~0.5 m/s and from trotting to galloping at ~0.7–0.8 m/s [10, 11]. Each of these gaits is associated with specific rhythmic patterns. Walking is the less regular of the gaits, with two or three limbs contacting the ground at each time and frequent stops. Trotting involves the rhythmic alternation of diagonal pairs of limbs (i.e., fore right/hind left followed by fore left/hind right) while in galloping both forelimbs contact the ground close in time, followed by both hindlimbs [9, 10]. The duration of each step cycle, defined as the time between successive floor contacts of a given foot, decreases with speed [11, 12]. Thus, the rates of the related forces acting on the body increase accordingly. Because of the left/right alternations in walking and trotting, the body experiences two cycles of rising and falling for each stride. Thus, the rate of stride forces acting on the body is twice that of stride itself [13, 14]. During the symmetrical motions of galloping these forces match the stride one to one, so their rate halves as the animal transitions to this gait from trotting [15].

The rhythms of locomotion and respiration can interact—a phenomenon known as locomotion-respiratory coupling [16]. In various species of mammals, including dogs, horses, and humans, respiration was found to synchronize with stride [15–19]. For nonhumans moving at stable moderate-to-high speeds the preferred locking is of one respiration cycle per stride (1 : 1 ratio) for symmetric and 2 : 1 for asymmetric gaits, although other stable ratios can be observed too. Both passive and active mechanisms have been proposed to explain this coupling. Rhythmic fluctuations in the loading of the lungs by forces associated with limb movements or displacement of inner organs may passively entrain respiration during locomotion [15–17]. A possible active mechanism has been described whereby activation of sensory input pathways from the limbs could entrain respiratory motor activity [20].

There is growing interest in the flexible coupling of sensorimotor rhythms characteristic of active rodent behavior [4, 8, 21–23]. To our knowledge, the interactions of these with the characteristic rhythmicity of locomotion have not yet been studied. In this work, we analyze recordings of head accelerometry, intranasal pressure, ultrasonic vocalizations (USVs), and video tracking to understand possible couplings between rhythmic behaviors during different modes of locomotion in freely behaving rats in a social context. We show evidence for phase coupling between the respiratory cycle and head/body movements associated with stride. The degree of coupling was, however, lower than what is usually reported for other quadrupeds. We show evidence suggesting that the emission of ultrasonic vocalizations disrupts the synchrony and propose that locomotion-respiration coupling is flexibly controlled during natural rat behavior.

## 2. Materials and Methods

### 2.1. Animal Subjects and Recording Sessions

This work combines recordings from two datasets. Most quantitative analysis comes from dataset 1, combining instantaneous speed, respiration, vocalizations, and head acceleration from 6 rats. To better understand the relationship between the acceleration signals and stride, we collected dataset 2 combining all of the above with high-speed video in 1 rat.

#### 2.1.1. Dataset 1

This dataset includes recordings from 6 Long Evans rats (Charles River): 2 females (ages 4.5–6.5 months) and 4 males (ages 2.5–5 months) interacting in male-female and male-male pairs. Total recording time for each rat was 46 and 28 minutes (females) and 74, 25, 12, and 10 minutes (males). Rats were implanted with intranasal cannulae and carried wireless headstage recording pressure from the cannula and head acceleration from a 3-axis accelerometer. The estrous cycle of females was controlled through ovariectomy and hormonal treatment and all recordings were made during estrus. Rats were held on an inverted light cycle and all recordings were carried out during the dark phase under infrared illumination. All procedures were approved by The Rockefeller University Institutional Animal Care and Use Committee (Protocol #09035).

#### 2.1.2. Dataset 2

This dataset includes recordings from 1 Long Evans male rat behaving alone or in the presence of a surgically devocalized female in hormonally induced estrus (both aged 5.0–6.1 months, Brain Institute animal facility). Respiration and head acceleration were recorded in the same way as for dataset 1 and detailed movements were captured with high-speed video under infrared illumination. All procedures were approved by the Animal Use Ethics Committee (Protocol #044/2014, CEUA/UFRN).

### 2.2. Surgical Procedures

Rats who underwent surgery were anesthetized with a combination of ketamine, xylazine, and atropine (i.m.: 100, 6, and 0.04 mg/kg, resp.). After surgery, buprenorphine (i.p.: 0.1 mg/kg) was administered as analgesic and enrofloxacin (i.p.: 20 mg/kg) as antibiotic. Animals recovered for at least one week before recordings.

As described in [4], the end of a thin 2 cm long stainless steel cannula (gauge 22) was implanted through the nasal bone. The cannula was bent to an S-shape for it to end above the temporal bone and secured with bone screws and dental acrylic. A ring magnet (R422; OD 6.35 mm, ID 3.18 mm; K&J Magnetics) was attached to the exposed end of the cannula to match an equivalent one secured to the pressure sensor in the wireless headstage. This allowed us to easily and safely secure the headstages on the rats' heads by using magnetic force only.

For bilateral ovariectomy, we made incisions through the skin and muscle posterior to the rib cage, through which we pulled the ovaries out with forceps. After clamping the uterine horns with hemostats and absorbable suture we proceeded to cut off the ovaries, suture the muscle with absorbable thread, and close the skin with suture clamps. For induction of estrus we injected females with estradiol benzoate (s.c.: 0.05 mg/kg) followed, 48 h later, by progesterone (s.c.: 2.5 mg/kg). Recordings were made 5–10 h after the administration of progesterone.

The female placed in the arena adjacent to the male for high-speed video recordings was devocalized so that all recorded vocalizations were from the male. We made a vertical incision on the skin of the neck (~3 cm) and divulsed the sternohyoid muscle. We then sectioned approximately 1 cm of the left laryngeal recurrent nerve [24].

### 2.3. Data Acquisition

We recorded animals in dataset 1 in an arena built with vertical gratings and split into two parallel linear tracks, 0.2 × 2.67 × 0.74 m (*W* × *L* × *H*) each, 0.15 m apart on the wide side. Sessions included one rat on each side of the arena. In dataset 2, the arena was identical except for its length of 3 m. Expanded methodological details will be published elsewhere.

#### 2.3.1. Video

We recorded video at 30 fps with two overhanging Logitech c920 webcams with infrared filters removed. We synchronized video with the other recorded signals with <1 frame precision through an infrared LED blinking in the visual field of the cameras controlled by the main data acquisition board. For dataset 2 we recorded video at 200 fps with a Point Grey FL3-U3-13Y3 M-C camera using FlyCapture2 software. We triggered frame acquisition with TTL pulses generated by the data acquisition board.

#### 2.3.2. Ultrasound

We recorded ultrasound with condenser microphones with nearly flat (±5 dB) response from 10 to 150 kHz (CM16/CMPA-5V, Avisoft Bioacoustics) digitized by a data acquisition board at 250 kHz sampling frequency (PCIe-6259 DAQ with BNC-2110 connector, National Instruments). For dataset 1 we recorded from 3 overhanging microphones distributed along the long axis of the arena. For dataset 2 we placed 1 microphone 10 cm from the floor pointing at the area within the visual field of the high-speed camera.

#### 2.3.3. Respiration and Accelerometry

**Figure 1:**
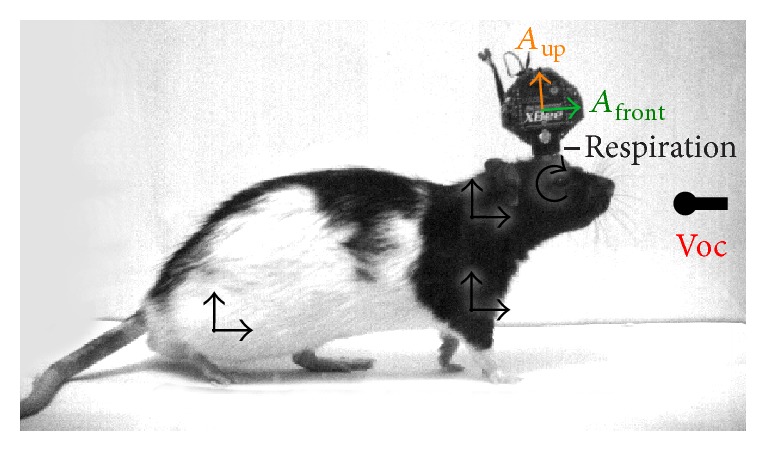
Recorded behavioral variables. Photograph of a male Long Evans rat, side view. The rat has a cannula with one end in the nasal cavity and the other over the cranium, with a ring magnet at its end. A wireless headstage carrying a pressure sensor and a 3-axis accelerometer is magnetically attached to the cannula. Throughout this work, respiration is intranasal pressure and *A*_front_ and *A*_up_ are the signals from the horizontal and vertical axes of the accelerometer. Ultrasonic vocalizations were recorded from ambient condenser microphones and instantaneous speed was measured from video tracking. Black arrows point to possible sources for the forces acting on the accelerometer: hindlimb and forelimb muscles, back/neck muscles, and changes in the decomposition of gravity from tilting of the head.

During recordings, we magnetically attached to the intranasal cannula a pressure sensor (24PCAFA6G, Honeywell) integrated into a custom-made wireless headstage based on the DIGI XBee module powered with 3 V from a Li-ion battery (weight 20–23 g, see Figure 1, schematics available on request). The headstage included a 3-axis accelerometer (ADXL335, Analog Devices) with signal filtered to 50 Hz 3 dB bandwidth with 0.1 *μ*F capacitors. Voltage outputs from this component are linearly proportional to acceleration (300 mV/g with 3 V supply voltage). The pressure and acceleration signals were transmitted with a sampling rate of 200 Hz each and digitized in synchrony with the ultrasound. The transmission imposed a 2-sample (10 ms) delay which was not corrected.

### 2.4. Data Preprocessing

We carried out all data preprocessing with custom-made routines in MATLAB (The Mathworks).

#### 2.4.1. Locomotion

We obtained the position of the rat in each video frame through a custom-made implementation of object tracking based on adaptive background subtraction. We smoothed the position by independently convolving the *X* and *Y* coordinates with a Gaussian window of full width at half maximum 0.25 s. We obtained the velocity vector as the first partial derivative of *X* and *Y* and computed instantaneous speed as its norm. We then segmented locomotion into progressing and lingering episodes implementing the methods developed in [25, 26]. We only considered progressions lasting at least 1 second and with a maximum speed of at least 0.2 m/s. We grouped all other segments together with interleaved arrests as “lingering” episodes.

#### 2.4.2. Ultrasonic Vocalizations

We thoroughly describe elsewhere the methodologies for automatically detecting ultrasonic vocalizations and assigning them to the emitting rat in a pair ([4] and to be published elsewhere). Briefly, detection involves finding times with low spectral entropy in the ultrasonic range and low noise in the sonic range at any of the three overhanging microphones. At times when USVs are detected, we compare the intranasal pressure of both rats and assign the USV to the one with characteristic constant pressure close to atmospheric values. We did not resolve cases of simultaneous vocalization from the two rats, so only one rat could be considered to be vocalizing at any given time. Because of this, when segregating respiration or acceleration cycles from one rat based on the emission of USVs, we discarded those cycles when the other was vocalizing, as those USVs could be obscuring calls from the analyzed rat. When needed, ultrasound was segmented by the sniffing cycle, such that all of the ultrasound emitted during a single exhalation is grouped as a USV.

We visually classified 100 randomly selected “50 kHz” USVs from each rat into four classes. “Flats” were identified as calls with little or no frequency modulation, with or without frequency jumps, “trills” as those of high frequency and frequency modulation, “flat-trills” as those combining the previous two elements, and “splits” as those with fundamental frequency momentarily jumping down to the 30–35 kHz range, typically with a visible second harmonic.

#### 2.4.3. Respiration and Accelerometry

Respiration is the intranasal pressure measurement and was left unfiltered, with atmospheric pressure baseline removed. We subtracted the baseline values from the acceleration signals with *A*_up_ pointing upwards (so its baseline included gravity force). Acceleration signals were then digitally bandpass filtered at 1–20 Hz, for example, figures and averaging at feet timestamps (Butterworth, order 3). When the animal tilts its head, gravity components should enter the signals. A tilt of 30° would change *A*_front_ by 4.9 and *A*_up_ by 1.3 m/s^2^. It was not possible to correct for these effects.

### 2.5. Data Analysis

#### 2.5.1. Frequency and Synchrony

Instantaneous respiratory rate was obtained as the inverse of the sniff cycle duration. Acceleration rates were obtained as the peak frequency in a Fourier spectrum of a 1-second window centered at each time point, previously convolved with a 0th-order Slepian taper. For synchrony analysis we bandpass filtered the data at 3–11 Hz with* eegfilt* [27] and obtained instantaneous phases from its Hilbert transform. For the acceleration signals, peak timestamps were defined as the times when the instantaneous phase crosses zero in the positive direction. For Phase Locking Value (PLV) calculation, we collected the instantaneous phases of a given signal (such as intranasal pressure) at a selected set of timestamps from another one (such as the peaks of *A*_front_ during trotting episodes) and constructed unity vectors with those phases as angles. PLV is the amplitude of the mean vector, such that if all have the same phase PLV = 1. To avoid effects of sample size in the PLV calculation [28] we used a subsampling strategy. From each group of phases, we calculated PLV as the mean PLV from 5000 random subsamples of fixed size 80, which was the smallest sample size in this work. With this sample size, the expected PLV for phases taken from a uniform distribution is 0.1. We statistically tested the departure of each distribution of phases from uniformity by applying the Rayleigh test, as implemented in CircStat [29]. *p* values lower than 1*e* ^−5^ were reported as *p* ≈ 0.

#### 2.5.2. Granger Causality

We analyzed Granger causality between intranasal pressure, emission of ultrasonic vocalizations, and *A*_front_ and *A*_up_ with tools from the MVGC toolbox [30]. A vocalization time series was constructed with ones at the times when the rat emitted ultrasound and zeros elsewhere. Data was sampled at 100 Hz and left unfiltered and 1-second episodes from each behavior mode were grouped as trials for multitrial analysis (each mode was analyzed independently). The model order for the autoregressive model was 20, equivalent to 200 ms. We measured band-limited G-causality in the 3–11 Hz range, so any contribution from slower (or faster) fluctuations was ignored (of note, cases with high overall causality always showed clear peaks in this range). We assessed significance empirically by trial shuffling, mixing the 4 variables across trials and repeating the analysis 500 times. Significance was established at alpha = 0.05 with Bonferroni correction for 12 comparisons. Arrows in Figure 8 have widths directly proportional to the obtained band-limited G-causality values (those not significant were removed). We confirmed these values were robust in that they depended little on sampling frequency, model order, exclusion of individual variables, or reduction in number of trials analyzed.

## 3. Results

We recorded locomotion, vocalizations, respiration, and head acceleration from 4 male and 2 female adult rats during free behavior (Figure 1). The position and instantaneous speed of the rats were obtained from video tracking (top view) under infrared light. Ultrasound was recorded from overhanging microphones and ultrasonic vocalizations were automatically detected from it. Each rat carried on its head a wireless headstage provided with a pressure sensor and a 3-axis accelerometer. The pressure sensor connected to an intranasal cannula to monitor the respiratory cycle. The accelerometer was positioned with one axis horizontal and directed to the front of the head “*A*_front_” and another one vertical and directed upwards “*A*_up_”. Acceleration of an object is linearly related to the force acting on it which, in the case of the head of the rat, can include contributions from muscles in the limbs and back/neck and gravity. The rats were behaving in pairs in an arena split in two long (2.7 m) corridors separated by wire gratings, one rat positioned at each side. The females were under hormonally induced estrus and the recordings analyzed include both male-female and male-male sessions. For most analyses we pooled together the data from all 6 rats (total recording time 195 minutes).

**Figure 2:**
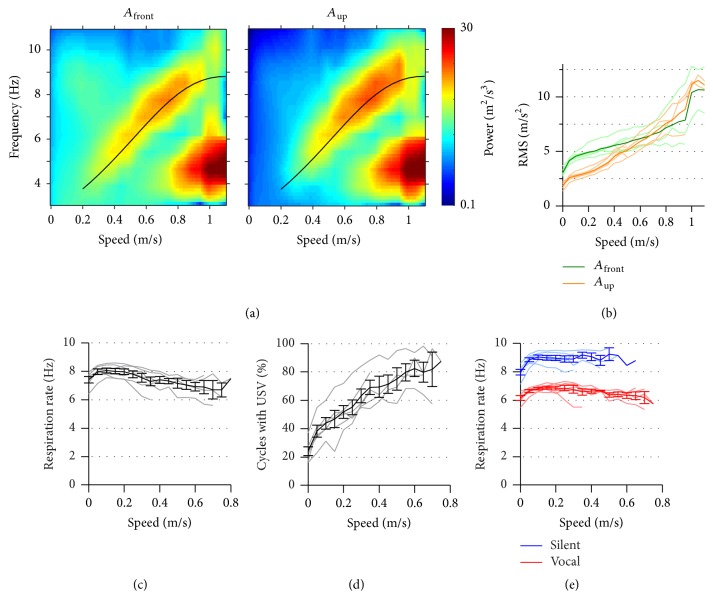
Distribution of respiration and head acceleration rates as a function of instantaneous speed. (a) Mean spectra of frontwards and upwards head acceleration (*A*_front_ and *A*_up_) for each range of rat instantaneous speed (color is log_10_ of power). Black line represents twice the stride rate measured by [11], fitted with a cubic spline. (b) Root mean square of *A*_front_ and *A*_up_ versus instantaneous speed. Thin lines represent the 6 individual rats and thick lines the mean across rats. (c) Mean instantaneous respiratory rate (1/cycle duration) versus mean instantaneous speed during each cycle. Gray lines are individual rats and black lines are mean ± s.e.m. across rats. (d) Percentage of respiratory cycles with ultrasonic vocalization emission versus rat instantaneous speed. Lines as in (c). (e) As in (c), segregating respiratory cycles with and without emission of vocalizations.

We sought to understand how respiration and emission of vocalizations couple with head movements and stride across different modes of behavior. As detailed above, each of these motor components can be rhythmic so we analyzed them as oscillatory signals and studied possible synchronizations between them. Based on previous knowledge and preliminary analysis, we looked for coupling in the 3–11 Hz, the typical range of rates for these variables during active behavior. We begin by analyzing how the rhythmicity of these signals varies with the instantaneous speed of the animal (Figure 2). We performed frequency analysis of head acceleration in a sliding 1 s window and plotted the mean spectrum for each instantaneous speed range (Figure 2(a)). Both *A*_front_ and *A*_up_ showed a similar behavior. At speeds under 0.2 m/s, signals were of low power and with no visible frequency peak. From there up to 0.8 m/s signals grow in power and show a peak in frequency rising from 4 to over 8 Hz. At higher speeds the rate of head movement halves, dropping to 4-5 Hz. The amplitude of the acceleration oscillation monotonically increased with speed (Figure 2(b)). At low speeds, *A*_front_ was larger than *A*_up_ but the latter grew larger for higher speeds. The halving of their rate at 0.8 m/s was matched by a further increase in their amplitude. At 0.2–0.8 m/s rats move with walking and trotting gaits, which involve alternation of left and right limbs [11, 14]. At these gaits the head bobs twice for each full stride, so that its rate should be twice the stride rate. Indeed, the peak rate of acceleration for this speed range matched published measurements of stride rate (Gillis and Biewener, 2001; black line in Figure 2(a)), suggesting that this signal was following forces related to locomotion. At higher speeds, rats are known to transition to a galloping gate, where there is no left/right alternation and the head completes one movement cycle per stride, which explains the observed rate halving. Thus, the head-mounted accelerometer signal can follow the stride cycle at intermediate-to-high speeds and report the transition from walking/trotting to galloping.

The respiration rate of awake rats has a large dynamic range from about 2 to 11 Hz, with a transition from passive breathing to active sniffing typically reported at 4-5 Hz [1–3]. If the respiratory and stride cycles were to consistently couple one to one, their rates should match across speeds. This was not the case in our recordings. We calculated instant respiratory rate as the inverse of the duration of each cycle and found its mean to be above 7 Hz for all speeds (Figure 2(c)), reflecting that the rats maintained high activity levels during the sessions. In detail, its mean rate moderately dropped with speed from 8 to 7 Hz. We reasoned that ultrasonic vocalizations of the “50 kHz” family could be causing the observed drop in respiratory rate, as we know that their emission increases during locomotion (details to be published elsewhere) and prolongs the respiratory cycle [4]. Indeed, the percentage of cycles with vocalizations increased with speed from 20 to 80% (Figure 2(d)). Cycles with USVs were about 2 Hz slower than those without them and the mean instantaneous rate of the silent respiratory cycles was above 8 Hz for all speeds where it could be measured (Figure 2(e)).

These exploratory analyses show that the behavior of the rats in the arena was not homogeneous. Instead, it interleaved moments of staying in place, with low vocal production and head acceleration, with locomotor behavior of varied stride rate and higher vocal output. We decided to segregate the behavior into 4 locomotion modes based on the recorded signals: staying in place, walking, trotting, and galloping. First, we segmented locomotion into episodes of “progressing,” moving between places, and “lingering,” staying in one location with only local movement [26, 31]. We noticed that during a single progression rats could use more than one gait. We thus kept from each progression only one second of data, centered at its peak speed. We found this to result in more homogenous locomotion events. We labeled as walking those progressions with maximum speed between 0.2 and 0.5 m/s. Those with speed above 0.5 m/s and peak *A*_up_ above 6 Hz (see Figure 2(a)) were labeled as trotting and those with speed above 0.8 m/s and peak *A*_up_ below 6 Hz as galloping. The transition speed between walking and trotting was chosen based on previous reports [11, 14]. We analyze now the possible synchronization between respiration and head/body movements for each of these 4 modes and postpone the analysis of their interplay with vocalizations for later. To better understand how the accelerometer signals relate to head/body movements we recorded all these variables together with high-speed video (side view, 200 frames per second) for one male rat behaving alone or in the presence of a neighboring devocalized female in estrus. Selected recorded episodes from this rat were classified as belonging to one of the 4 described locomotion modes by matching detailed observation of the videos to previous descriptions of gait [10].

### 3.1. Sniffing Behavior

**Figure 3:**
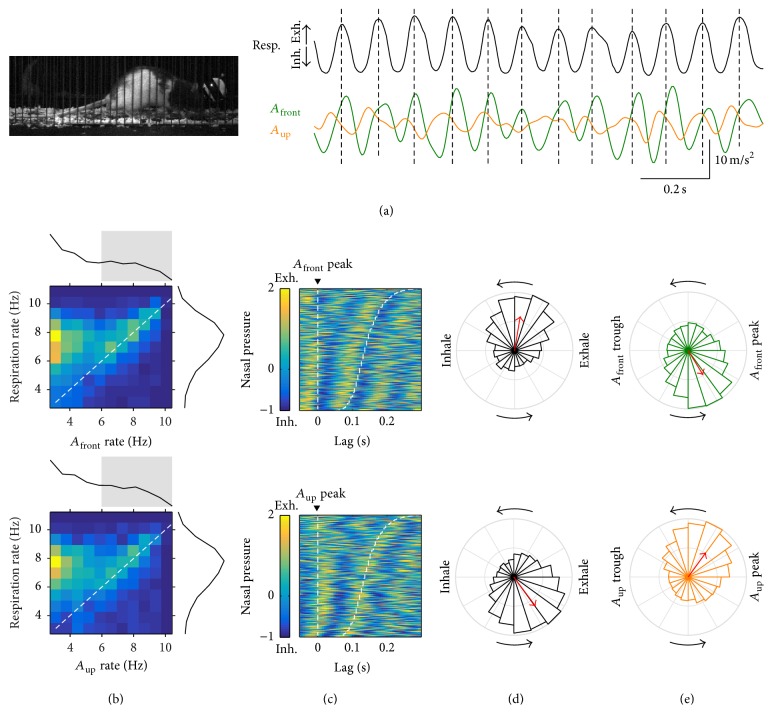
Sniffing behavior. (a) Example of sniffing behavior. Left: video frame of the rat sniffing while staying in place. Right: respiration (intranasal pressure, a.u.) and frontwards (*A*_front_) and upwards (*A*_up_) head acceleration. The falling phase of the *A*_front_ cycle matches with head approach, while the rising phase matches head withdrawals. This is consistent with acceleration being the derivative of velocity. The rat did not vocalize during this period. Dashed lines mark the time of exhalation peaks. (b) Joint distribution of mean respiratory rate and head acceleration rate for each sniffing episode with marginal distributions on the sides (in (b)–(e), top is *A*_front_ and bottom is *A*_up_). Warm colors represent highest concentration of values (see color gradient in (c)). Dashed line follows the diagonal. Gray shading in the acceleration marginal distribution represents the cases included in the analysis in (c)–(e). (c) Alignment of respiration to all acceleration peaks detected in the analyzed sniffing episodes. Each row corresponds to a different cycle, sorted from top to bottom by acceleration cycle duration. Dashed lines mark the beginning and end of each acceleration cycle. (d) Phase locking of the respiratory cycle to the acceleration peaks. (e) Phase locking of the acceleration cycle to the peak of exhalation. As a guide for interpreting phase plots, the top panel in (d) shows that, at the times of *A*_front_ peaks, respiration was typically transitioning from the peak of exhalation to the peak of inhalation, with a PLV of 0.29, while top panel in (e) shows that at the times of peak exhalation *A*_front_ was typically on its rising phase with a PLV of 0.24.

Rats engage in various behaviors while staying in place such as grooming, rearing, and sniffing. We sought to study periods of sniffing behavior, when the rats are known to coordinate fast respiration with head movements and whisking [1].  Figure 3(a) shows an example of this, where respiration and acceleration are apparently synchronized and match approach and withdrawal movements of the head (Supplementary Video 1 in Supplementary Material available online at http://dx.doi.org/10.1155/2016/4065073). To detect from the large dataset periods of sniffing behavior we obtained a smoothed measure of respiratory rate and collected nonoverlapping 1-second episodes around peaks of at least 3 Hz (total 2675, discarding episodes with very low acceleration power). For each episode, we calculated mean rates for respiration, *A*_front_, and *A*_up_ and obtained their joint distribution (Figure 3(b)). Interestingly, those with fast rhythmic acceleration (>6 Hz) matched their rates with the respiration. We selected those episodes for further analysis. We first obtained timestamps for all *A*_front_ and *A*_up_ peaks within those episodes and aligned the intranasal pressure signal to them. As shown in Figure 3(c), *A*_front_ and *A*_up_ peaks coincided with the transitions from exhalation to inhalation and from inhalation to exhalation, respectively. It also shows that the timing of the following acceleration peak matches that of a later transition in respiration, so that the duration of head movement and respiration cycles is matched. To quantify this phase synchrony, we obtained the distribution of instant respiratory cycle phases at the time of acceleration peaks (Figure 3(d)). Respiratory phases at the time of *A*_front_ peaks clustered around the midtime between the exhalation peak and the following inhalation peak with Phase Locking Value = 0.29 (*p* ≈ 0, *N* = 9494 cycles). Respiratory phases at the time of *A*_up_ peaks clustered past the midtime between the inhalation peak and the following exhalation peak with PLV = 0.31 (*p* ≈ 0, *N* = 9247 cycles). If two oscillations are symmetrically coupled, phase locking of any one to the peaks of the other should be of similar magnitude. Indeed, at the time of exhalation peaks, *A*_front_ and *A*_up_ were, respectively, clustered at their rising and falling phases (Figure 3(e), PLV = 0.24 and 0.26, *p* ≈ 0 for both, and *N* = 8374 and 8505 cycles). Note that the rising phase of *A*_front_ and the falling phase of *A*_up_ both correspond to the withdrawal of the head (head moving back and up). Thus, it was possible to detect in the freely behaving rat events of sniffing as periods of fast coordinated rhythmicity between respiration and head acceleration, with phase relationships that match those previously described: head withdrawal during exhalations and head approach during inspirations [1].

### 3.2. Walking

**Figure 4:**
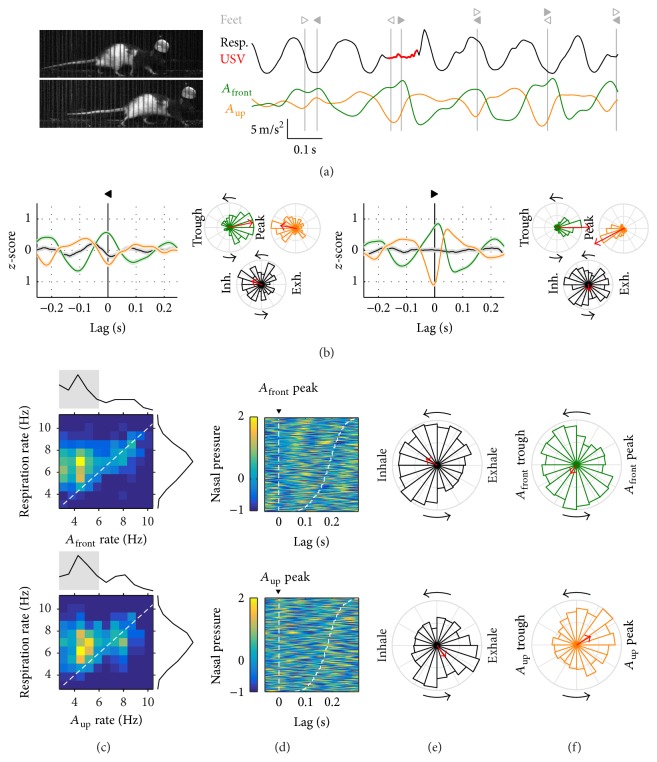
Walking. (a) Example of walking behavior. Left: video frames coinciding with right hindlimb (top) and right forelimb (bottom) contact. Right: respiration (intranasal pressure, a.u.) and frontwards (*A*_front_) and upwards (*A*_up_) head acceleration. Times with emission of ultrasonic vocalizations are painted in red on the respiration signal. Vertical gray lines mark the time of footsteps (right/left triangles: fore- and hindlimbs; filled and open symbols: left and right limbs). Max. speed at this progression was 0.46 m/s. (b) Alignment of respiration and acceleration signals to footsteps from hind- (left panels) and forelimbs (right panels). For each, left plot is mean ± s.e.m. traces for respiration and accelerations at the footstep times. Right phase plots are distribution of *A*_front_, *A*_up_, and respiration cycle phases at the same footstep times. Colors are as in (a). Red arrows represent mean phase with the amplitude of the vector proportional to Phase Locking Value (a full radius equals PLV = 0.5). As a guide for interpreting these graphs, the phase plots for hindlimb footsteps show that at those times the *A*_front_ oscillation was typically near its peak, with a PLV of 0.46; *A*_up_ was near its trough with a PLV of 0.30 while respiration was less synchronized, with PLV 0.16 and mean phase near the peak of inhalation. (c) Joint distribution of mean respiratory rate and head acceleration rate for each walking episode with marginal distributions on the sides (top: *A*_front_; bottom: *A*_up_). Warm colors represent highest concentration of values (see color gradient in (d)). Dashed line follows the diagonal. Gray shading in the acceleration marginal distribution represents the cases included in the analysis in (d)–(f). (d) Alignment of respiration to all acceleration peaks detected in the analyzed walking progressions. Each row corresponds to a different cycle, sorted from top to bottom by acceleration cycle duration. Dashed lines mark the beginning and end of each acceleration cycle. (e) Phase locking of the respiratory cycle to the acceleration peaks. (f) Phase locking of the acceleration cycle to the peak of exhalation.

We now consider those progressions labeled as walking (maximum speed between 0.2 and 0.5 m/s). Observation of high-speed videos evidenced that rats would sometimes engage in sniffing behavior while walking, such that the head acceleration signals would be dominated by head approach/withdrawal and not by the stride movements (see below for the large dataset). We will now focus on walking progressions with both acceleration rates below 6 Hz (37 episodes) and leave the analysis of sniffing while walking for Supplementary Figure 1.  Figure 4(a) shows an example of walking gait under high-speed video (Supplementary Video 2). We quantified the relationship between stride and head acceleration during walking by aligning the latter to the times when the front or hind feet touched the ground (the beginning of the stance part of each step cycle) (Figure 4(b)). The onset of both front and hind feet stance coincided with the peak of *A*_front_, that is, the time of maximal frontal force acting on the head. At these times *A*_up_ was at its trough, when vertical force was maximal in the downwards direction. Quantification showed that forces acting on the head were more tightly synchronized to the front feet (PLV for *A*_front_ and *A*_up_ at feet timestamps = 0.59 and 0.63, *p* ≈ 0 for both, and *N* = 181 steps) than to the hind ones (PLV = 0.46 and 0.30, *p* ≈ 0 for both, and *N* = 174 steps). Alignment of the respiratory cycle to these step times was low (PLV at the times of hind and front feet = 0.16 and 0.08 and *p* = 0.02 and 0.9). We analyzed 409 progressions labeled as walking in the large dataset. As for lingering episodes, the joint distribution of *A*_front_ and *A*_up_ rates with respiratory rate revealed two populations: one with acceleration rates above 6 Hz matching the fast respiratory rates and one with acceleration rates centered on 4-5 Hz (Figure 4(c)). Feet cycles during walking are too long for the observed high rates of head movement to be matching the stride (see Figure 2(a) and [11]). These episodes represented instead cases of sniffing behavior while walking (Supplementary Figure 1). We continue here the analysis for those with slower acceleration rates (*N* = 202), which match the expected stride rates for walking and in our high-speed video observations aligned with the step cycles. Alignment of respiration cycles to acceleration peaks during walking was lower than that observed for sniffing behavior (Figures 4(d) and 4(e), compared to Figures 3(c) and 3(d)), particularly so for *A*_front_ (PLVs for respiration at peaks of *A*_front_ and *A*_up_ = 0.13 and 0.16, *p* ≈ 0 for both, and *N* = 1421 and 1340 cycles). Synchronization of acceleration signals to exhalation peaks was also lower, although less so for *A*_up_ (Figure 4(f), PLVs for *A*_front_ and *A*_up_ at exhalation peaks = 0.12 and 0.17, *p* < 0.0001 for both, and *N* = 1796 and 1658 cycles). Note that the favored respiration phase at the time of *A*_up_ peaks was the transition from inhalation to exhalation, similarly to what was observed during sniffing. In summary, our rats exhibited partial synchrony between respiration and head/body movements while walking.

### 3.3. Trotting

**Figure 5:**
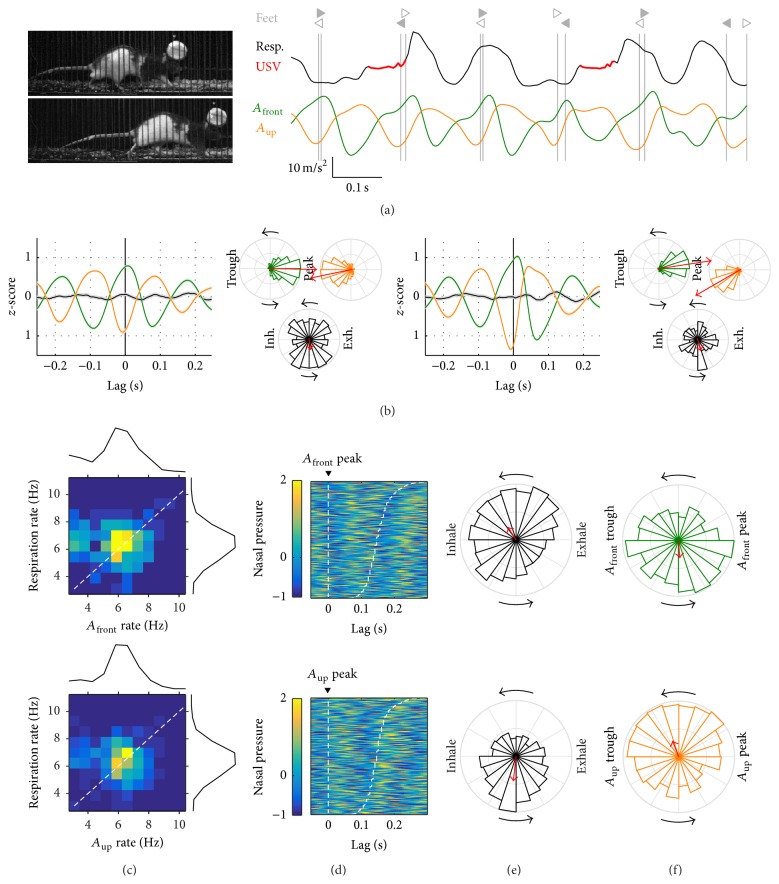
Trotting. (a) Example of trotting behavior. Left: video frames coinciding with right hindlimb (top) and right forelimb (bottom) contact. Right: respiration (intranasal pressure, a.u.) and frontwards (*A*_front_) and upwards (*A*_up_) head acceleration. Symbols as in Figure 4(a). Max. speed at this progression was 0.61 m/s. (b) Alignment of respiration and acceleration signals to footsteps from hind- (left panels) and forelimbs (right panels) as in Figure 4(b). (c) Joint distribution of mean respiratory rate and head acceleration rate for each walking episode with marginal distributions on the sides (top: *A*_front_; bottom: *A*_up_, as in Figure 4(c)). (d) Alignment of respiration to all acceleration peaks detected in the analyzed trotting progressions (as in Figure 4(d)). (e) Phase locking of the respiratory cycle to the acceleration peaks. (f) Phase locking of the acceleration cycle to the peak of exhalation.

Figure 5(a) shows an example of trotting under high-speed video recording (Supplementary Video 3). During trotting, a given diagonal pair of feet (i.e., front left and hind right) contacts the floor close in time, alternating with the other diagonal pair. We characterized the synchrony between stride and head acceleration while trotting as we did before for walking (Figure 5(b), 91 episodes). Forces acting on the head aligned with front and hind feet at similar phases, with *A*_front_ being maximal and *A*_up_ minimal at the times when the feet touched the ground. Synchronization was, however, visibly tighter during trotting with respect to hind feet (PLV for *A*_front_ and *A*_up_ at feet timestamps = 0.66 and 0.59, *p* ≈ 0 for both, and *N* = 419 steps) and even more so for the front feet (PLV for *A*_front_ and *A*_up_ at feet timestamps = 0.81 and 0.80, *p* ≈ 0 for both, and *N* = 405 steps). Again, the respiratory cycles were poorly aligned to these steps (PLV at the times of hind and front feet = 0.11 and 0.12 and *p* = 0.052 and 0.024). The joint distribution of *A*_front_ and *A*_up_ rates with respiratory rate for progressions labeled as trotting in the large dataset (*N* = 267 episodes) revealed a homogeneous population with head movement rates in the 5–7 Hz range (Figure 5(c)), consistent with the expected rhythmicity of stride (see Figure 2(a)). Interestingly, mean respiration rates also matched this range. Despite this similarity in mean rates, respiration again showed only partial synchronization with forces acting on the head (Figure 5(d)). The strongest synchrony was that of respiration phase to the peak of vertical acceleration, while phase locking to the peak of the horizontal one was lower (PLVs for respiration at peaks of *A*_front_ and *A*_up_ = 0.14 and 0.23, *p* ≈ 0 for both, and *N* = 1795 and 1738 cycles). The favored respiration phase at the time of *A*_up_ peaks was the transition from inhalation to exhalation, similarly to what we observed during sniffing and walking. Synchronization of acceleration signals to exhalation peaks was partial too (Figure 5(f), PLVs for *A*_front_ and *A*_up_ at exhalation peaks = 0.15 and 0.16, *p* ≈ 0 for both, and *N* = 1795 cycles).

### 3.4. Galloping

**Figure 6:**
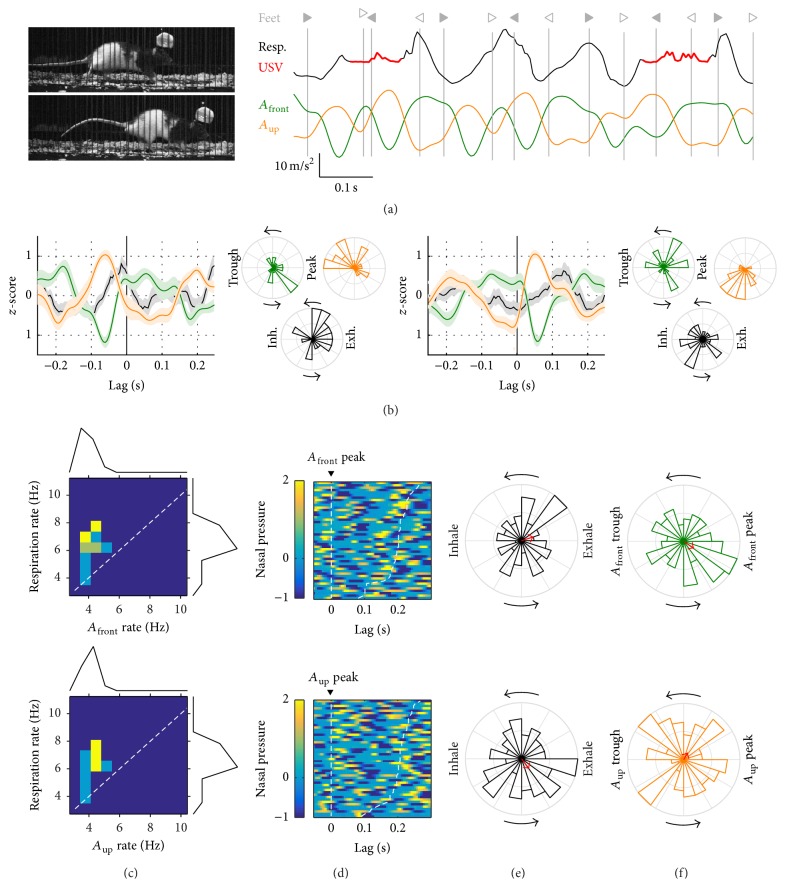
Galloping. (a) Example of galloping behavior. Left: video frames coinciding with right forelimb (top) and right hindlimb (bottom) contact. Right: respiration (intranasal pressure, a.u.) and frontwards (*A*_front_) and upwards (*A*_up_) head acceleration. Symbols as in Figure 4(a). Max. speed at this progression was 0.72 m/s. (b) Alignment of respiration and acceleration signals to footsteps from hind- (left panels) and forelimbs (right panels) as in Figure 4(b). We did not calculate PLVs because of the low number of samples. (c) Joint distribution of mean respiratory rate and head acceleration rate for each walking episode with marginal distributions on the sides (top: *A*_front_; bottom: *A*_up_, as in Figure 4(c)). (d) Alignment of respiration to all acceleration peaks detected in the analyzed trotting progressions (as in Figure 4(d)). (e) Phase locking of the respiratory cycle to the acceleration peaks. (f) Phase locking of the acceleration cycle to the peak of exhalation.

We detected only a few cases of galloping in our recordings: 5 in the dataset with high-speed video and 15 in the larger one.  Figure 6(a) shows an example of this gait (Supplementary Video 4). During galloping, both front feet land closely in time, followed by both hind feet. Aligning of head acceleration to the feet timestamps suggests that peaks in the vertical forces acting on the head occur about 50 ms prior to the landing of the hind feet and 50 ms past the landing of the front ones (Figure 6(b), *N* for hind and front feet = 23 and 28). Phase locking of head acceleration and respiration at the feet timestamps was unclear. The 15 progressions labeled as galloping in the large dataset formed a homogeneous population with low rate of head movements (4-5 Hz, Figure 6(c)). Examination of respiration cycles aligned to peaks of head acceleration revealed no apparent synchrony between the signals (Figure 6(d)). Accordingly, Phase Locking Values for galloping were the lowest across all behavioral modes studied, both for alignment of respiration to head movements (Figure 6(e), PLVs for respiration at peaks of *A*_front_ and *A*_up_ = 0.1 and 0.11, *p* = 0.43 and 0.39, and *N* = 80 and 80 cycles) and of head movements to respiration (Figure 6(f), PLVs for *A*_front_ and *A*_up_ at exhalation peaks = 0.11 and 0.07, *p* = 0.35 and 0.76, and *N* = 104 cycles). Note that the magnitude of the Phase Locking Value we obtain does not depend on the sample size (see Methods).

### 3.5. Disruption of Coupling between Respiration and Head/Body Movements during Vocalizations

**Figure 7:**
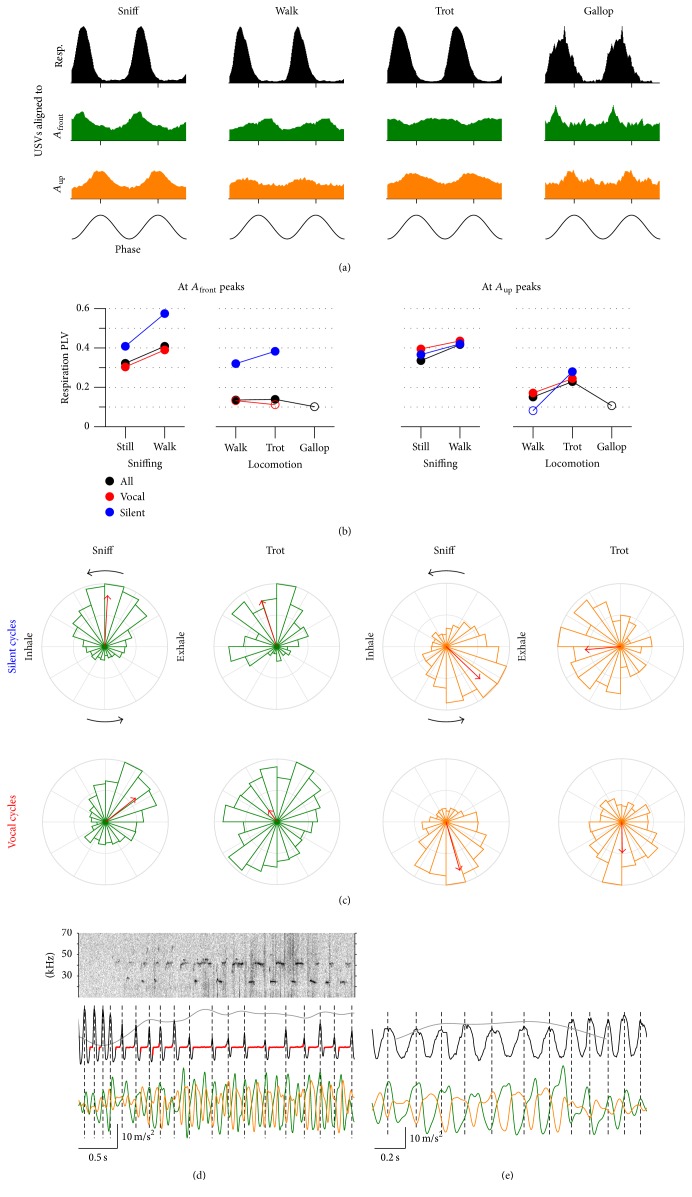
Disruption of coupling between respiration and head/body movements during vocalizations. (a) Distribution of emitted ultrasound with the phase of the respiration and acceleration oscillations for each behavior mode. The vertical axes represent how many times the rats emitted ultrasound at each phase of each oscillation. The histogram covers two cycles for clarity. As a guide to read these plots, note that the rats almost never emitted ultrasound while the respiration was between its peak exhalation and the following peak inhalation. A total number of USVs detected were 1089 (sniffing), 500 (walking), 938 (trotting), and 54 (galloping). Note that phases between the respiration and acceleration are not matched, since each one was aligned to its own peak. (b) Phase Locking Values for respiration at the times of *A*_front_ (left) and *A*_up_ (right) peaks for all acceleration cycles (black), only those that did not coincide with emission of vocalizations (blue), or only those that did (red). We included in sniffing those during lingering (detailed in Figure 3) and those during walking (detailed in Supplementary Figure 1). There were not enough cycles during galloping episodes to segregate by vocalizations. (c) Phase locking of respiration to *A*_front_ (left) and *A*_up_ (right) peaks during trotting and sniffing. Values with no significant phase locking are depicted with open symbols (Rayleigh test, alpha = 0.05/26 = 0.002). (d) Example of trotting with high vocal production from a male rat. Top: sonogram of the recorded ultrasound (dark is high power). Bottom: respiration, vocalization times, and frontwards (*A*_front_) and upwards (*A*_up_) head acceleration (colors as in Figure 4(a)). Gray solid line is speed (max. 0.79 m/s) and dashed lines mark the time of exhalation peaks. The rat is doing fast respiration and then starts a progression while emitting long vocalizations of the “split” class, which were found to coincide with fast progressions in a separate set of recordings (to be published elsewhere). Note that during long vocalizations respiration appears decoupled with head acceleration. (e) Example of trotting without vocalizations for the male rat under high-speed video (max. speed 0.5 m/s). Note that *A*_up_ peaks appear to align with the transition from inhalations to exhalations.

Rats in our social arena were emitting high rates of ultrasonic vocalizations (mean 2.0, range 1.3–2.5 calls/sec). All calls were of the “50 kHz” family, including “flats” (38 ± 5%), “trills” (24 ± 8%), “flat-trills” (28 ± 5%), and “splits” (10 ± 4%, mean ± s.e.m., *N* = 6 rats; see Methods). Rat ultrasonic vocalizations are known to be intimately linked to the respiratory cycle [4]. Specifically, vocalizations begin at the transition from inhalation to exhalation, prolong the exhalation phase, and are followed by a final silent exhalation phase before the onset of the next inhalation. Since our analysis revealed some degree of synchronization between respiration and head acceleration during sniffing, walking, and trotting behavior, it is to be expected that the emission of vocalizations will be correlated to the phase of the acceleration signals as well. Indeed, ultrasound emission is modulated by the phase of *A*_front_ and *A*_up_ oscillations for these behaviors (Figure 7(a)). Note, however, that this modulation is weaker than that observed against the phase of the respiratory cycle (Figure 7(a)). Since the emission of vocalizations instantly disrupts the ongoing respiratory rhythm by delaying the following inhalation, we reasoned this could affect synchronization between respiration and other behavioral variables. We quantified the effects of vocalizations on the synchrony between respiration and head acceleration by segregating cycles with and without ultrasound emission from the rat. Each acceleration cycle was defined between two successive peaks and was considered “vocal” if during those times any ultrasound was detected from that rat or “silent” otherwise. We collected the phases of the respiratory rhythm at the end of each of these cycles and calculated their PLVs (Figure 7(b)). Interestingly, respiration was consistently better synchronized to silent *A*_front_ cycles than to vocal ones. Synchrony between respiration and *A*_up_ peaks was not much affected by the recent history of vocalization. Of note, silent *A*_front_ peaks showed better synchrony with respiration than *A*_up_ ones, opposite to what was observed when considering all cycles. A possible explanation for vocalizations having little effect on the synchrony between *A*_up_ and respiration is that *A*_up_ peaks typically align with the onset of the exhalation phase such that if a vocalization starts, it can only affect the synchrony at the next cycle, typically over 150 ms later. *A*_front_ peaks, on the other hand, typically align with the offset of the exhalation, a phase of the respiratory cycle that will be variably delayed by vocalizations of different duration, thus blurring the synchrony.  Figure 7(c) details the distribution of respiration phase at acceleration peaks for silent versus vocal cycles during sniffing and trotting behavior. Overall, these results suggest that vocalizations can instantly disrupt the synchronization of respiration to head/body movements. This analysis is limited in that it can only reveal immediate and short-lived disruptions of synchrony by vocalizations. From the observation of examples with long vocalizations it seems clear that head and body movements can maintain their ongoing rhythms during vocal emission, effectively decoupling themselves from respiration (Figure 7(d)). Cases with intermediate-to-high speed and no vocalizations were rare, preventing us from quantifying coupling in those conditions.  Figure 7(e) shows an example from a male during a silent slow trot (while being recorded alone in the arena). In this and other silent examples, it appeared that better synchrony arose between respiration and head/body movements, with exhalations following the *A*_up_ peaks and thus matching the preferred phase relationships in our dataset.

### 3.6. Causality

**Figure 8:**
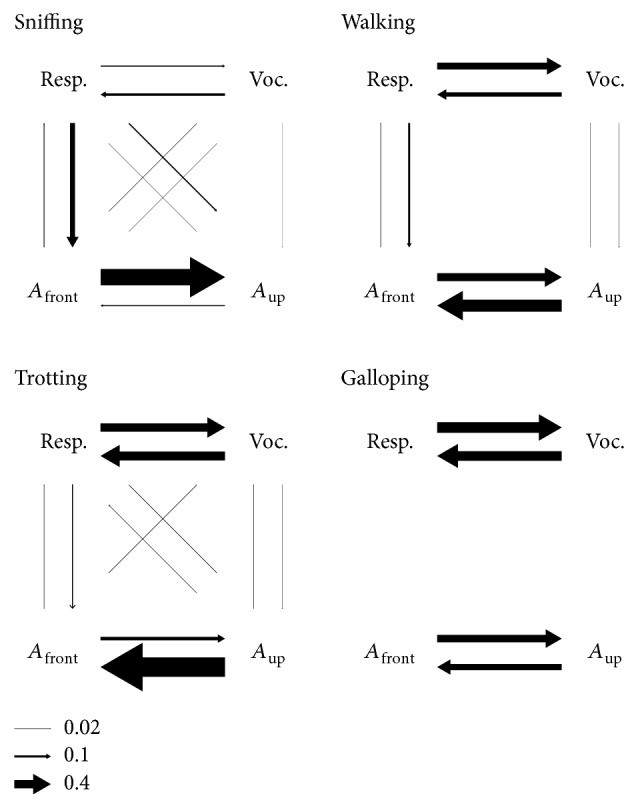
Causality. Granger causality between intranasal pressure, emission of ultrasonic vocalizations, and head acceleration for each behavioral mode. Causality was assessed in the 3–11 Hz frequency range. Arrows represent significant causality from one variable to another, with line width proportional to its magnitude. Reference arrows are shown on the bottom.

As we have seen, all recorded behavioral variables show some degree of coupling to all others. This makes it difficult to understand if there exist hierarchies between them, such as locomotion forces entraining the respiratory cycle. We looked for possible directionalities in the coupling by measuring Granger causality between four time series: intranasal pressure, ultrasonic vocalization emission, and the two axes of head acceleration. Given a set of variables (*X*, *Y*, *Z*,…) evolving in time, *Y* is said to* G-cause X* if knowing the recent past of all the variables gives a better prediction of the present of *X* than just knowing the recent past of all variables except *Y*. In this case, the past of *Y* contains information about the present of *X* that was not present in the other variables (including *X*) and so it is interpreted that changes in *Y cause* changes in *X*. We measured causality in our set of 4 variables for each behavioral mode considering 200 ms of the recent past, equivalent to one oscillation cycle at 5 Hz (Figure 8). Overall, forces acting on the head had very low or no predictive value towards emission of vocalizations and vice versa. During sniffing, the respiration signal predicted the frontal force on the head and, in turn, this one strongly predicted the vertical forces (note that these effects are not at all symmetrical). Respiration and vocalizations show only moderate degrees of mutual causality during sniffing, possibly because vocal production is low while staying in place. During locomotion, when forces acting on the head synchronized with limb movements, causalities were low between these and respiration in both directions, with the strongest among these being the respiratory cycle predicting frontal force. The respiratory cycle was predictive of vocalization emission and vice versa, consistent with the known bidirectional mechanistic relationship between both. Horizontal and vertical acceleration of the head were mutually causal, with *A*_up_ being particularly strong in predicting *A*_front_ during trotting. Altogether, these results are compatible with a hierarchy of couplings where respiration and vocalizations are intimately bound while their coupling with head/body movements is secondary.

## 4. Discussion

We simultaneously monitored respiration, vocalizations, head acceleration, and locomotion in rats freely behaving in a social context. We focused on behaviors where these motor components oscillate and analyzed their detailed synchrony—or lack of it. The biggest challenge in our study came from the heterogeneity of relatively unconstrained natural behavior. From our observations, most behavioral patterns in the rat do not last longer than one or a few seconds, such that consistent but short-lived relationships between variables could be easily missed. Building on solid existing literature describing the different components of rat behavior, we devised ways of automatically parsing active behavior into four modes based on instantaneous speed and rhythmicity of respiration and head/body movements: sniffing (in place or while walking), walking, trotting, and galloping.

Sniffing is a characteristic sensorimotor behavior displayed by rats during exploration. It combines fast (5–11 Hz) respiration with head and whisker movements, all of these coordinated into a common cycle [1, 8]. We detected putative sniffing bouts as periods of fast (>6 Hz) respiration and head movements without fast locomotion. These episodes adhered in detail to previous descriptions of sniffing, with consistent phase relationships: head withdrawal during exhalations and head approach during inspirations. We have no measure of whisking in our large dataset but the rat under high-speed video recording was actively whisking during sniffing bouts.

Horizontal and vertical forces measured by the head-mounted accelerometer were consistently in antiphase during rhythmic behaviors, with some small but consistent phase differences during different behavioral modes. Although these two variables are not independent, it is possible that they are dominated by forces from different sets of muscles. It is interesting that respiration aligned better to *A*_up_ peaks than to *A*_front_ ones during trotting and walking. Causality analysis suggests a hierarchy between horizontal and vertical forces during these gaits, such that the latter entrains the former. During walking and trotting, the time of first contact of both hind and front feet with the floor coincided with the maximum of frontward and downward force acting on the head. We cannot tell from our data which components of the forces are made by limb or neck muscles. Comparing our results with measurements of ground reaction forces, it seems that the resultant forces acting on the head have some phase differences with those made by the limbs. The onset of the stance phase during trotting coincides with the trough of vertical force both measured on the ground and on the head [14]. However, our horizontal force is almost in antiphase with the vertical component, while the horizontal ground reaction forces peak leads the vertical ones by about a quarter cycle. Regardless of this, our results show that acceleration measured at the head can be used to track the limb cycle during locomotion.

Our results point to a flexible coupling between respiration and locomotion forces during spontaneous rat behavior. It is clear that head acceleration and respiration are synchronized to some degree during walking and trotting, such that there is a larger probability of exhalations beginning around the peak of the vertical component. This phase relationship is consistent with that observed in dogs and rabbits [17, 19, 32]. The degree of synchrony we found was, however, lower than those reported for other mammals. Note that synchrony was worse when aligning respiration phase directly to the footsteps of the rat recorded under high-speed video. It could thus be that respiration couples more to head movements during stride than to stride itself. During walking, we cannot rule out that some of the synchrony reflects brief contributions of sniffing behavior. In trotting cases when synchrony was apparent, locking was 1 : 1 between acceleration and respiration cycles (equivalent to 2 respiration cycles per stride, as found for dogs and large opossums [17, 19, 33]). Despite respiratory rate being approximately twice the head movement rate at low speeds, we found no evidence for sustained 2 : 1 locking mode, as observed in other small mammals [33], such that 2 breaths coincide with each acceleration cycle. We cannot rule out that this happens for some scattered cycles. Rats were vocalizing at high rates during locomotion, in up to 80% of the sniff cycles at their fastest speeds. Our analysis suggests that emission of a vocalization results in an immediate perturbation in the coupling between respiration and stride, consistent with the known instantaneous prolonging of the exhalation phase [3, 4]. Contraction of trunk musculature during vocal exhalation could decouple respiration from stride forces acting on the lungs. Complementarily, central mechanisms could be blocking proprioceptive influences on respiratory pattern generators. It has been proposed that rodent ultrasound emission is a byproduct of explosive exhalations caused by loading of the thoracic cavity upon forelimb contact during locomotion [34]. Our results disprove this strict causal relationship, as modulation of vocal emission by stride is incomplete and rats can maintain vocal rhythms independent of ongoing locomotion even at high speeds. We do not rule out, however, that stride mechanics could be modulating the detailed timing and properties of vocalizations through their partial coupling with respiration—although such effects were not supported by our causality estimation. We could not analyze broader effects of vocal rates on coupling simply because our rats were rarely walking fast, trotting, or galloping without vocalizing. In the few cases of silent trotting, obtained from one male rat behaving in isolation, we observed reasonable synchrony between respiration and stride. We would need to find natural nonsocial conditions where rats are sufficiently motivated to trot and gallop to extend our study to spontaneous silent locomotion.

We acknowledge some limitations in our work. To be able to track body movements during free behavior we mounted on the head a ~20 g wireless headstage (about 5% of body weight). This is common practice in freely moving electrophysiology studies, and rats seem to quickly get habituated to carrying weight on the head. Our case is potentially more problematic since we are directly measuring acceleration at the head, which depends on force and mass. Rats carrying our headstage engaged in many behaviors with apparent normality. For those behaviors which we analyzed quantitatively, their properties matched previous descriptions: sniffing occurred at the expected rates and phase relationships of respiration and head approach/withdrawal; head acceleration rates matched twice the published stride rates for walking and trotting and once that rate for galloping; peak vertical forces align with the onset of the exhalation. Despite this, it is possible that detailed magnitudes and phases are affected by the headstage weight. We could not extract a clear transition from fast walking to trotting from the data without high-speed video, so we set a threshold at max. progression speed of 0.5 m/s to classify them. It remains unclear to us whether a clear-cut difference between both gaits exists in the rat or rather one smoothly turns into the other as speed increases. There were few instances of galloping in both our datasets, but enough to observe that no obvious locking between respiration and locomotion appears as the rats switch to this gait. Younger estrus females and males should be studied to better sample this behavior. Because not all rats reached fast trotting and galloping, we pooled together data from all of them for most analysis. The fundamental mechanical properties studied in this work likely generalize to the rat population, but we cannot make inferences about their variability across individuals or their correlation with variables such as sex and age.

## 5. Conclusions

Rats moving freely in space exhibited only partial coupling between the respiratory and stride cycles. Our results clearly differed from the tight cycle-to-cycle synchrony frequently found for other quadruped mammals [35]. We do not know of other works measuring this synchronization in the rat, so we wonder whether this contrast is due to species differences, scaling of coupling with body mass, or differences in the behavioral settings. Most measurements of locomotor-respiratory coupling make efforts to homogenize the locomotor behavior by using treadmills or guiding the animals into running straight lines at stable speeds. These conditions deliberately minimize perturbations that could interfere with the coupling. The rats in our arena were rapidly changing speed, direction, and gait of locomotion and perturbing the respiratory rhythm through emission of vocalizations. We propose that locomotion-respiratory coupling is not a requirement of the system but rather a flexible entrainment that can be overridden by other physiological or behavioral needs such as vocal communication. Two directions should be followed to disambiguate between species and behavioral setting: measure coupling in rats running silently at stable speeds and remeasure it in other mammals during rich natural behavior.

Restricting animal behavior has proven an invaluable tool in understanding its building blocks and mechanisms. New insights can be gained by complementing this with studies where behavior is less bounded and thus closer to the conditions under which it evolved. This approach however poses big challenges on the recording and analysis side. This is particularly true for small animals like rats and mice, for whom behavioral variables evolve fast. A way to compensate for the loss of homogeneity is to develop reliable ways to parse free behavior into stereotyped modes. Additional challenges stem from the intercorrelated nature of these variables. For example, one can believe that a given physiological measure (like neuronal activity at a given brain structure) is partially correlated with the phase of the respiratory cycle when, in fact, this was actually secondary to a tight association between that measure and stride. Understanding the magnitude of these correlations at each behavioral mode will aid in avoiding these pitfalls. We acknowledge that missing variables could even be confounding the correlations presented in this work.

## Supplementary Material

Supplementary Material includes one figure and four videos. Supplementary Figure 1 analyzes the coupling of respiration to head movements while the rats perform sniffing behavior while walking. Supplementary videos 1 to 4 are examples episodes of sniffing, walking, trotting and galloping behavior respectively.

## References

[1] Welker W. I. (1964). Analysis of sniffing of the albino rat. *Behaviour*.

[2] Wachowiak M. (2011). All in a sniff: olfaction as a model for active sensing. *Neuron*.

[3] Hegoburu C., Shionoya K., Garcia S., Messaoudi B., Thévenet M., Mouly A.-M. (2011). The RUB cage: respiration-ultrasonic vocalizations-behavior acquisition setup for assessing emotional memory in rats. *Frontiers in Behavioral Neuroscience*.

[4] Sirotin Y. B., Costa M. E., Laplagne D. A. (2014). Rodent ultrasonic vocalizations are bound to active sniffing behavior. *Frontiers in Behavioral Neuroscience*.

[5] MacLarnon A. M., Hewitt G. P. (1999). The evolution of human speech: the role of enhanced breathing control. *American Journal of Physical Anthropology*.

[6] Assini R., Sirotin Y. B., Laplagne D. A. (2013). Rapid triggering of vocalizations following social interactions. *Current Biology*.

[7] James J. E., De Burgh Daly M. (1972). Reflex respiratory and cardiovascular effects of stimulation of receptors in the nose of the dog. *The Journal of Physiology*.

[8] Moore J. D., Deschênes M., Furuta T. (2013). Hierarchy of orofacial rhythms revealed through whisking and breathing. *Nature*.

[9] Grillner S. (1975). Locomotion in vertebrates: central mechanisms and reflex interaction. *Physiological Reviews*.

[10] Locomotion M. G. (2004). *The Behavior of the Laboratory Rat*.

[11] Gillis G. B., Biewener A. A. (2001). Hindlimb muscle function in relation to speed and gait: in vivo patterns of strain and activation in a hip and knee extensor of the rat (*Rattus norvegicus*). *Journal of Experimental Biology*.

[12] Mendes C. S., Bartos I., Márka Z., Akay T., Márka S., Mann R. S. (2015). Quantification of gait parameters in freely walking rodents. *BMC Biology*.

[13] Bobbert M. F., Gómez Álvarez C. B., van Weeren P. R., Roepstorff L., Weishaupt M. A. (2007). Validation of vertical ground reaction forces on individual limbs calculated from kinematics of horse locomotion. *Journal of Experimental Biology*.

[14] Muir G. D., Whishaw I. Q. (2000). Red nucleus lesions impair overground locomotion in rats: a kinetic analysis. *European Journal of Neuroscience*.

[15] Young I. S., Alexander R., Woakes A. J., Butler P. J., Anderson L. (1992). The synchronization of ventilation and locomotion in horses (*Equus caballus*). *Journal of Experimental Biology*.

[16] Bramble D. M., Carrier D. R. (1983). Running and breathing in mammals. *Science*.

[17] Bramble D. M., Jenkins F. A. (1993). Mammalian locomotor-respiratory integration: implications for diaphragmatic and pulmonary design. *Science*.

[18] Baudinette R. V., Gannon B. J., Runciman W. B., Wells S., Love J. B. (1987). Do cardiorespiratory frequencies show entrainment with hopping in the tammar wallaby?. *Journal of Experimental Biology*.

[19] Carrier D. R. (1996). Function of the intercostal muscles in trotting dogs: ventilation or locomotion?. *Journal of Experimental Biology*.

[20] Morin D., Viala D. (2002). Coordinations of locomotor and respiratory rhythms in vitro are critically dependent on hindlimb sensory inputs. *The Journal of Neuroscience*.

[21] Moore J. D., Kleinfeld D., Wang F. (2014). How the brainstem controls orofacial behaviors comprised of rhythmic actions. *Trends in Neurosciences*.

[22] Ranade S., Hangy B., Kepecs A. (2013). Multiple modes of phase locking between sniffing and whisking during active exploration. *The Journal of Neuroscience*.

[23] Rao R. P., Mielke F., Bobrov E., Brecht M. (2014). Vocalization-whisking coordination and multisensory integration of social signals in rat auditory cortex. *eLife*.

[24] Roberts L. H. (1975). Evidence for the laryngeal source of ultrasonic and audible cries of rodents. *Journal of Zoology*.

[25] Drai D., Benjamini Y., Golani I. (2000). Statistical discrimination of natural modes of motion in rat exploratory behavior. *Journal of Neuroscience Methods*.

[26] Hen I., Sakov A., Kafkafi N., Golani I., Benjamini Y. (2004). The dynamics of spatial behavior: how can robust smoothing techniques help?. *Journal of Neuroscience Methods*.

[27] Delorme A., Makeig S. (2004). EEGLAB: an open source toolbox for analysis of single-trial EEG dynamics including independent component analysis. *Journal of Neuroscience Methods*.

[28] Scheffer-Teixeira R., Tort A. B. L. (2016). Lack of evidence for cross-frequency phase-phase coupling between theta and gamma oscillations in the hippocampus. *bioRxiv*.

[29] Berens P. (2009). CircStat: a MATLAB toolbox for circular statistics. *Journal of Statistical Software*.

[30] Barnett L., Seth A. K. (2014). The MVGC multivariate Granger causality toolbox: a new approach to Granger-causal inference. *Journal of Neuroscience Methods*.

[31] Golani I., Benjamini Y., Eilam D. (1993). Stopping behavior: constraints on exploration in rats (*Rattus norvegicus*). *Behavioural Brain Research*.

[32] Simons R. S. (1999). Running, breathing and visceral motion in the domestic rabbit (*Oryctolagus cuniculus*): testing visceral displacement hypotheses. *Journal of Experimental Biology*.

[33] Reilly S. M., White T. D. (2009). Breathing with your belly: abdominal exhalation, loco-ventilatory integration and size constraints on locomotion in small mammals. *Zoology*.

[34] Blumberg M. S. (1992). Rodent ultrasonic short calls: locomotion, biomechanics, and communication. *Journal of Comparative Psychology*.

[35] Boggs D. F. (2002). Interactions between locomotion and ventilation in tetrapods. *Comparative Biochemistry and Physiology Part A: Molecular & Integrative Physiology*.

