# Supplementary material for: Flexible Coupling of Respiration and Vocalizations with Locomotion and Head Movements in the Freely Behaving Rat
Source: Neural Plast. 2016 Jul 25;2016:4065073. doi: 10.1155/2016/4065073 (PMC4976156; doi:10.1155/2016/4065073)
Supplement: Supplementary file 1 — Supplementary Material includes one figure and four videos. Supplementary Figure 1 analyzes the coupling of respiration to head movements while the rats perform sniffing behavior while walking. Supplementary videos 1 to 4 are examples episodes of sniffing, walking, trotting and galloping behavior respectively. [file 4065073.f1.zip › 4065073 Mat/Supplementary Material - Flexible coupling of respiration and vocalizations with locomotion and head movements in the freely behaving rat.pdf]

Flexible coupling of respiration and vocalizations with locomotion and head movements in the freely behaving rat

Alves, Joseph Andrews†, Boerner, Barbara Ciralli †, Laplagne, Diego Andrés*

Brain Institute, Federal University of Rio Grande do Norte, Natal, Brazil

* Correspondence: Diego A. Laplagne, dlaplagne@neuro.ufrn.br
† These authors contributed equally to this work

Supplementary material

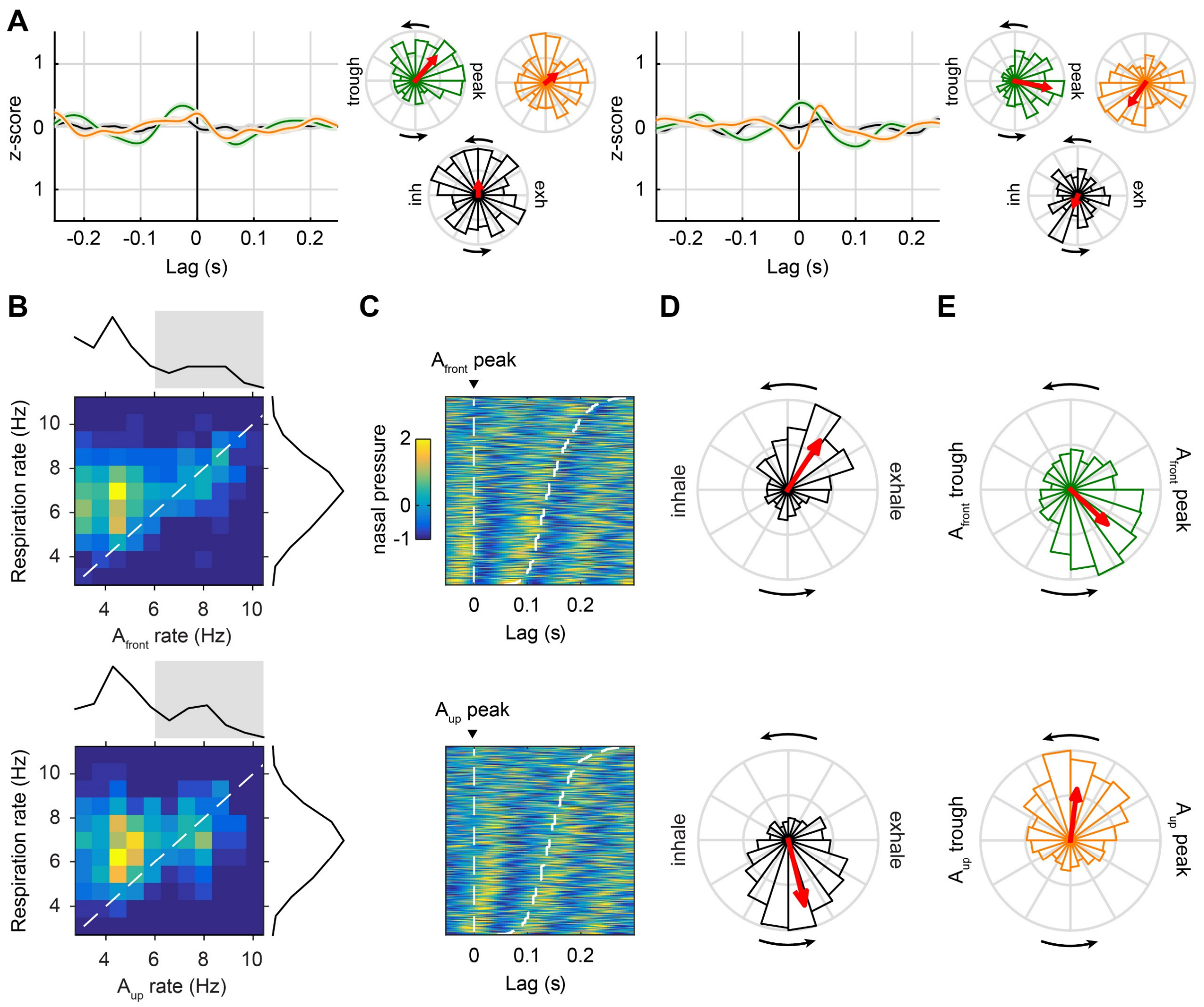

Supplementary Figure 1. Sniffing while walking

A. Alignment of respiration and acceleration signals to footsteps from hind (left panels) and forelimbs (right panels) as in Figure 4B (PLV for A_{front} and A_{up} at hindfeet timestamps = 0.27 and 0.13, $p \approx 0$ and 0.04, N = 287 steps; PLV for A_{front} and A_{up} at fore feet timestamps = 0.30 and 0.19, $p \approx 0$ and < 0.0001, N = 305 steps; PLV for respiration at the times of hind and fore feet = 0.09 and 0.10, p = 0.5 and 0.3).

Notice the lower synchrony of steps with head acceleration during sniffing while walking than during walking (Figure 4B). **B.** Joint distribution of mean respiratory rate and head acceleration rate for each sniffing-while-walking episode with marginal distributions on the sides (top: A_{front}; bottom: A_{up}, as in Figure 4C). Gray shading in the acceleration marginal distribution represent the cases included in the analysis in C-E. **C.** Alignment of respiration to all acceleration peaks detected in the analyzed sniffing-while-walking bouts (As in Figure 4D). **D.** Phase locking of the respiratory cycle to the acceleration peaks (PLVs for respiration at peaks of A_{front} and A_{up} = 0.33 and 0.38, $p \approx 0$ for both, N = 1151 and 1087 cycles). **E.** Phase locking of the acceleration cycles to the peak of exhalation (PLVs for A_{front} and A_{up} at exhalation peaks = 0.28 and 0.29, $p \approx 0$ for both, N = 1151 and 1289 cycles). Notice the better alignment of all signals during sniffing while walking when compared to walking (Figure 4D-F). Of note, coupling was also higher than during sniffing while staying in place (Figure 3C-E).

Supplementary videos

Example episodes of sniffing behavior, walking, trotting and galloping (Supplementary Videos 1, 2, 3 and 4). Top: frames from high-speed video (200 fps). The headstage is seen protected with polystyrene foam. Bottom: simultaneous recordings of intranasal pressure (‘Sniff’, blue), horizontal acceleration A_{front} (‘Front/Back’, dark orange) and vertical acceleration A_{up} (‘Up/Down’, orange). The time axis spans 2 seconds and time 0 corresponds to the video frame being shown. Respiratory cycles with emission of vocalizations show a period of near-zero pressure following the end of the inhalation phase, some of them with high-frequency fluctuations. Videos are slowed down 20 times.
